# Rising prevalence of HIV infection and associated risk factors among young Thai Men in 2018

**DOI:** 10.1038/s41598-021-87474-7

**Published:** 2021-04-08

**Authors:** Julius Eleazar dC. Jose, Boonsub Sakboonyarat, Mathirut Mungthin, Kenrad E. Nelson, Ram Rangsin

**Affiliations:** 1 Graduate Program in Biomedical Science, Faculty of Allied Health Sciences, Thammasat University, Rangsit Campus, Klong Luang, Pathum Thani Thailand; 2 Department of Medical Technology, Faculty of Pharmacy, University of Santo Tomas, Manila, Philippines; 3 Department of Military and Community Medicine, Phramongkutklao College of Medicine, Bangkok, 10400 Thailand; 4 Department of Pharmacology, Phramongkutklao College of Medicine, Bangkok, 10400 Thailand; 5 Department of Epidemiology, Johns Hopkins University Bloomberg School of Public Health, Baltimore, MD USA

**Keywords:** HIV infections, Risk factors, Epidemiology

## Abstract

The prevalence of HIV among young Thai men stabilized at 0.5% from 2005 to 2011. A cross-sectional study was conducted among the male army conscripts in 2018 at 36 military training units nationwide. All new conscripts in each selected unit were invited to participate in the study. Questionnaires were used to determine risk factors to HIV infection that had been developed from related risk factors studies among young Thai men. Among 4629 participants, 44 (1.0%) HIV positive individuals were identified. The proportion subject reporting a history of sex with another man was 10.1%. The prevalence of HIV infection among men who have sex with men (MSM) was 4.0%. The proportion of consistent condom use with a male partner was 39.7%. The risk factors of HIV infection included having sex with another man, history of sexually transmitted infection and history of sex in exchange for gifts/money. Only 1.4% of MSM used pre-exposure prophylaxis (PrEP). HIV prevention programs including PrEP in Thailand should be emphasized among MSM in both rural and urban settings.

## Introduction

According to the UNAIDS DATA 2020, at the end of 2019, approximately 470,000 adults were living with HIV and more than one half were men. In the same year, a total of 5400 new adult HIV cases were identified and more than two thirds comprised men^1^. The surveillance among newly recruited conscripts has long provided significant information on the status of the epidemic and the behavioral data provide evaluation of existing measures as well as a reference for relevant and up to date preventive measures^2^.

In 2015, a national behavioral risk factor study was conducted among a sample of young Thai men aged from 17 to 29 years that were inducted in the Royal Thai Army (RTA) during the induction rounds from November 2005 until May 2009^3^. This was followed in May 2011 by similar results^2^. The prevalence of HIV infection among young Thai men was 0.5% between 2005 and 2011^2,3^. Additionally, sexual behaviors including men who have sex with other men (MSM) were significant risk factors for HIV infection among young Thai men^3^.

Similarly, the recent report in the US demonstrated that the national HIV prevalence among MSM had a 4.5-fold increase in the past century^4^. The related study also reported that the prevalence of HIV among MSM in several countries was higher than the prevalence of that among men^5^. Therefore, HIV transmission among MSM is considered to be an important driver of the global HIV epidemic.

In 2017, the National AIDS Prevention and Alleviation Committee reported that more than 50% of new cases occurred among those aged 15–24 years and 40% were MSM. Sexual transmission remained the primary mode of transmission of which 41.8% were between MSM, 31.3% among married heterosexual couples and 13.3% among nonregular partners^6^.

The current study aimed to determine prevalence of HIV infection and risk behaviors among the sample of newly inducted army conscripts who entered military service in November 2018 to examine the current epidemiology among young Thai men. The information from a sample of young men in Thailand provided useful information in monitoring the epidemic and implementing effective preventive measures.

## Methods

### Study participants

The study population was enrolled as previously described^2,7^. The young Thai men aged 21 years, selected by a lottery system, rendered military service to the RTA. The conscription was conducted annually in April, at the district level of their home province. A small subset of men, found incapable of rendering military service, were excluded including those with severe sickness, transgender women and individuals who participated in alternative military service including the Thai Reserve Officer Training Corps Student program^7^. The exemption process does not exclude young men with asymptomatic HIV or exclude individuals based on their sexual orientation or drug use. Legal sanctions and penalties are given to those who do not participate in the selection process or do not render military service without a valid exception. Since 2001, men both younger and older than 21 years were accepted to volunteer without going through the lottery system. These men enter military service either in May or November^3^. All new RTA conscripts were voluntarily invited to participate in the national HIV surveillance program. This includes serological testing for HIV and a short demographics questionnaire that does not include any behavioral risk factor questions^3,7^.

### Data collection

In Thailand, 37 RTA hospitals are located in five regions i.e., north, central, south, northeast, and Bangkok nationwide. Each RTA hospital provided medical care for the responsible basic military training units in the area. The basic military training units were selected using stratified cluster sampling method. A cluster of basic military training units under the supervision of one RTA hospital in each region was selected. Of 323 basic military training units nationwide, 36 basic military training units located in five provinces of five regions; nine units in Chiang Mai (North), seven units in Lopburi (Central), nine units in Songkhla (South), six units in Ubon Ratchathani (Northeast) and five units in Bangkok were selected to be the study sites. All new conscripts in each selected unit were invited to participate in the study. After providing written informed consent, participants were given a self-administered standardized questionnaire that included demographic characteristics and behavioral risk factors for HIV infection. This questionnaire was deployed before the release of the HIV test result to minimize response bias. Anonymity was ensured by using unique codes that could only be decoded by the respective data management personnel of the project. The questionnaires were used to determine risk factors to HIV infection that had been developed from related risk factors studies among young Thai men^7^. Men having sex with men status was defined as the lifetime sexual activity of man having sex with another man and not as the identity expressed by the person.

### Statistical analysis

The individual responses to the self-administered risk factor survey were encoded to a computer- based program with their HIV sero-status added at the end of the process. Data were analyzed using IBM SPSS Statistics for Windows, Version 23.0. Mean and standard deviation were computed to describe continuous data while percentage was used to describe categorical data. Chi-square or Fisher’s exact test was calculated to determine the association of the categorical data while independent t-test was calculated to determine associations of the continuous variables. The odds ratios (OR) and 95% confidence intervals (CI) of demographic and behavioral variables associated with HIV seroprevalence were evaluated using univariate analysis. A multivariate logistic regression model was used to determine the independent effects of significant risk factors for HIV infection. Statistical significance was determined using a *p*-value less than 0.05.

### Ethics consideration

The study protocol was approved by the Institutional Review Boards of the Royal Thai Army Medical Department and the Ethics Subcommittee of Thammasat University (approval number: S009q/61). Written informed consent was obtained from the participants after they read the information sheet and signed the consent form. Identified HIV positive conscripts were given post-test counseling and necessary treatment was provided following Thai national guidelines.

## Results

### Demographic characteristics

A total of 38,779 conscripts were inducted in November 2018 nationwide. Of these, 38,255 (98.6%) participated in the national surveillance program for HIV. Of 4786 conscripts from the selected 36 basic military training units in five provinces, 4629 (96.72%) participants enrolled in the present study. The demographics and behavioral profiles of the enrolled participants before conscription are presented in Table 1. The average age of the participants was 21.6 ± 1.2 years. In all, 1284 (28.8%) men came from the northern region, 1314 (29.4%) came from the northeastern region, 564 (12.6%) came from the central region, 979 (21.9%) came from the southern region, and 324 (7.3%) came from Bangkok. Of all participants, 58.2% of young men resided in an urban area two years before conscription. The participants obtained bachelor’s degree or higher accounted for 10.7%. In all, 10.1% (95%CI; 9.1–11.0%) of individuals reported history of sex with another man. In terms of sexual preference or sexual desire, 97.6% preferred to have sex with females only while 1.3% preferred to have sex with exclusively with males and 1.1% preferred to have sex with both males and females. Among young men, 5.5% of men knew PrEP and only 0.4% used PrEP. The proportion of history of previous HIV testing among participants was 25.6%. The proportion of consistent condom use with a FSW was 83.7, while that with a male partner was 39.7%.

**Table 1:** Demographics and behavioral profiles of the participants before induction.

Characteristics (n = 4629)	n (%)
**Age (years)**	
Mean ± SD	21.6 ± 1.2
**Living pattern**	
With parents	3366 (75.1)
With lover/wife	673 (15.0)
With relatives and friends	339 (7.5)
Alone	103 (2.3)
**Region of residence 2 years before induction**	
North	1284 (28.8)
Northeast	1314 (29.4)
Central	564 (12.6)
South	979 (21.9)
Bangkok	324 (7.3)
**Place of residence**	
Urban	1896 (58.2)
Rural	1361 (41.8)
**Occupation**	
Employee	2195 (54.6)
Student	628 (15.6)
Agriculture	609 (15.2)
Unemployed	587 (14.6)
**Marital status**	
Single	3403 (75.0)
Married	1137 (25.0)
**Educational attainment**	
No formal to grade 6	906 (19.8)
Grade 7 to grade 12	2165 (47.3)
Vocational and diploma	1104 (22.2)
Bachelor’s degree or higher	492 (10.7)
**History of injecting drug use**	220 (5.1)
**History of non-injecting drug use**	1996 (44.8)
**History of previous HIV testing (lifetime)**	986 (25.6)
**History of blood transfusion**	228 (5.0)
**History of circumcision**	625 (14.1)
**History of sexual intercourse**	4169 (91.0)
**Average age of first sex (years)**	16.6 ± 2.1
**History of sex with a female sex worker**	889 (21.4)
**Number of lifetime sexual partner**	
0–1	1035 (23.6)
2–5	1504 (34.3)
≥ 6	1847 (42.1)
Median (min–max)	5 (0–78)
**History of sex with another man**	429 (10.1)
**Sexual experience (lifetime)**	
Exclusively heterosexual	3660 (89.6)
Bisexual	361 (8.8)
Exclusively homosexual	66 (1.6)
**Sexual preference/desire**	
Female only	4455 (97.6)
Male only	58 (1.3)
Both male and female	50 (1.1)
**History of sexually transmitted infection**	231 (5.7)
**History of sex in exchange for gifts/money**	231 (5.4)
**History of sexual coercion**	162 (3.8)
**Knows about PrEP**	251 (5.5)
**Uses PrEP**	16 (0.4)
**Knows about PEP**	155 (3.4)
**Uses PEP**	23 (0.5)
**History of consistent condom use (n/N)**	
With female sex workers	340/406 (83.7)
With a male partner	94/237 (39.7)
**HIV infected (cases)**	44 (0.95)

*PrEP* pre-exposure prophylaxis, *PEP* post-exposure prophylaxis.

We had the opportunity to identify MSM based on their reported homosexual activity. The demographics and behavioral profiles of MSM compared with non-MSM are shown in Table 2.

**Table 2:** Demographics and behavioral profiles of identified men who have sex with men (MSM) and non-MSM among the participants before induction.

Characteristics	MSM (%)	Non-MSM (%)	*p* value
	n = 429	n = 3826	
**Age (years)**			0.161^a^
Mean (± SD)	21.6 (± 1.3)	21.6 (± 1.2)	
**Living pattern**			0.273^b^
With parents	286 (69.9)	2764 (74.1)	
With lover/wife	73 (17.8)	602 (16.1)	
With relatives or friends	37 (9.0)	281 (7.5)	
Alone	13 (3.2)	85 (2.3)	
**Region of residence 2 years before induction**			0.296^b^
North	108 (26.7)	1073 (28.9)	
Northeast	123 (30.4)	1114 (30.0)	
Central	43 (10.6)	488 (13.1)	
South	97 (24.0)	778 (20.9)	
Bangkok	34 (8.4)	262 (7.1)	
**Place of residence**			0.492^c^
Urban	162 (55.9)	1576 (58.0)	
Rural	128 (44.1)	1139 (42.0)	
**Marital status**			
Single	302 (72.4)	2754 (73.0)	0.816^c^
Married	115 (27.6)	1018 (27.0)	
**Occupation**			0.672^b^
Employee	200 (54.9)	1866 (56.0)	
Student	50 (13.7)	507 (15.2)	
Agricultural	61 (16.8)	489 (14.7)	
Unemployed	53 (14.6)	470 (14.1)	
**Education attainment**			< 0.001^b^
No formal to grade 6	113 (26.5)	732 (19.3)	
Grade 7 to grade 12	208 (48.8)	1811 (47.7)	
Vocational and diploma	57 (13.4)	866 (22.8)	
Bachelor’s degree or higher	48 (11.3)	385 (10.1)	
**History of injecting drug use**			< 0.001^c^
Yes	47 (11.8)	162 (4.5)	
No	353 (88.2)	3448 (95.5)	
**History of non-injecting drug use**			0.002^c^
Yes	220 (53.8)	1687 (45.6)	
No	189 (46.2)	2016 (54.4)	
**History of previous HIV testing (lifetime)**			
Yes	118 (36.6)	812 (25.1)	< 0.001^b^
No	204 (63.4)	2425 (74.9)	
**Age (years) at first sexual intercourse**			0.260^a^
Mean (± SD)	16.8 (± 2.4)	16.6 (± 2.1)	
**History of sex with a female sex worker**			< 0.001^c^
Yes	131 (31.6)	753 (20.3)	
No	284 (68.4)	2958 (79.7)	
**Number of lifetime sex partner**			< 0.001^a^
Mean ± SD	27.7 (± 33.8)	20.9 (± 30.6)	
Median (range)	6.0 (0–78)	5.0 (0–78)	
**Sexual experience (lifetime)**			< 0.001^b^
Exclusive heterosexual	–	3660 (100.0)	
Exclusive homosexual	66 (15.5)	–	
Bisexual	361 (84.5)	–	
**Sexual preference/desire**			< 0.001^c^
Female only	339 (81.1)	3782 (99.7)	
Male only	47 (11.2)	2 (0.1)	
Both male and female	32 (7.7)	11 (0.3)	
**History of sexual transmitted infection**			< 0.001^c^
Yes	42 (11.0)	188 (5.1)	
No	341 (89.0)	3480 (94.9)	
**History of sex in exchange for gifts/money**			< 0.001^c^
Yes	60 (14.5)	169 (4.5)	
No	354 (85.5)	3606 (95.5)	
**History of sexual coercion**			< 0.001^c^
Yes	34 (8.1)	128 (3.4)	
No	385 (91.9)	3649 (96.6)	
**Knowledge of PrEP**	64 (15.5)	154 (4.1)	< 0.001^c^
**Use of PrEP**	6 (1.4)	8 (0.2)	0.001^c^
**Knowledge of PEP**	41 (9.8)	102 (2.7)	< 0.001^c^
**Use of PEP**	4 (1.0)	18 (0.5)	0.159^c^
**HIV infected cases**	17 (4.0)	26 (0.7)	< 0.001^c^

*PrEP* pre-exposure prophylaxis, *PEP* post-exposure prophylaxis.

^a^*p* value for comparison of mean of characteristic between group (independent sample t-test).

^b^*p* value for comparison of proportion of characteristics between group (Chi-square test).

^c^*p* value for comparison of proportion of characteristics between group (Fisher’s exact test).

The proportion of MSM participants residing in rural communities was comparable with non-MSM participants. Those who reported MSM activity had a higher proportion of risk factors for HIV than of those non-MSM participants, i.e., injecting and non-injecting drug use, number of life time sexual partners, history of sex with a FSW, history of STI, history of sex in exchange for gifts/money, and history of sexual coercion. Among those MSM participants, the proportions of reported exclusive homosexual and bisexual experiences were 15.5 and 84.5%, respectively. However, in terms of sexual preference or sexual desire among MSM participants, 81.1% preferred to have sex with females only while 11.2% preferred to have sex exclusively with males and 7.7% preferred to have sex with both males and females. The proportion of participants reported history of previous HIV testing among MSM and non-MSM was 36.6 and 25.1%, respectively. Among MSM participants, 15.5% knew PrEP and only 1.4% used PrEP.

### Prevalence of HIV infection among young Thai men in 2018

Of the 38,255 new RTA conscripts participating in the national HIV surveillance program, we found that 336 (0.9%, 95% CI: 0.8–1.0%) men were HIV positive. Of the enrolled young Thai men, 4629 (96.7%) participated in the study and 44 (1.0%, 95% CI: 0.7–1.3%) HIV positive cases were identified. The prevalence of HIV infection among young Thai men by region of residence two years before induction were 1.5, 1.3, 1.2, 1,1, 0.4 and 0.4% in Bangkok, northeast, lower north, upper north, south, and central, respectively. The prevalence of HIV infection among young Thai men residing in urban and rural areas was 1.1 and 0.8%, respectively.

**Figure 1:**
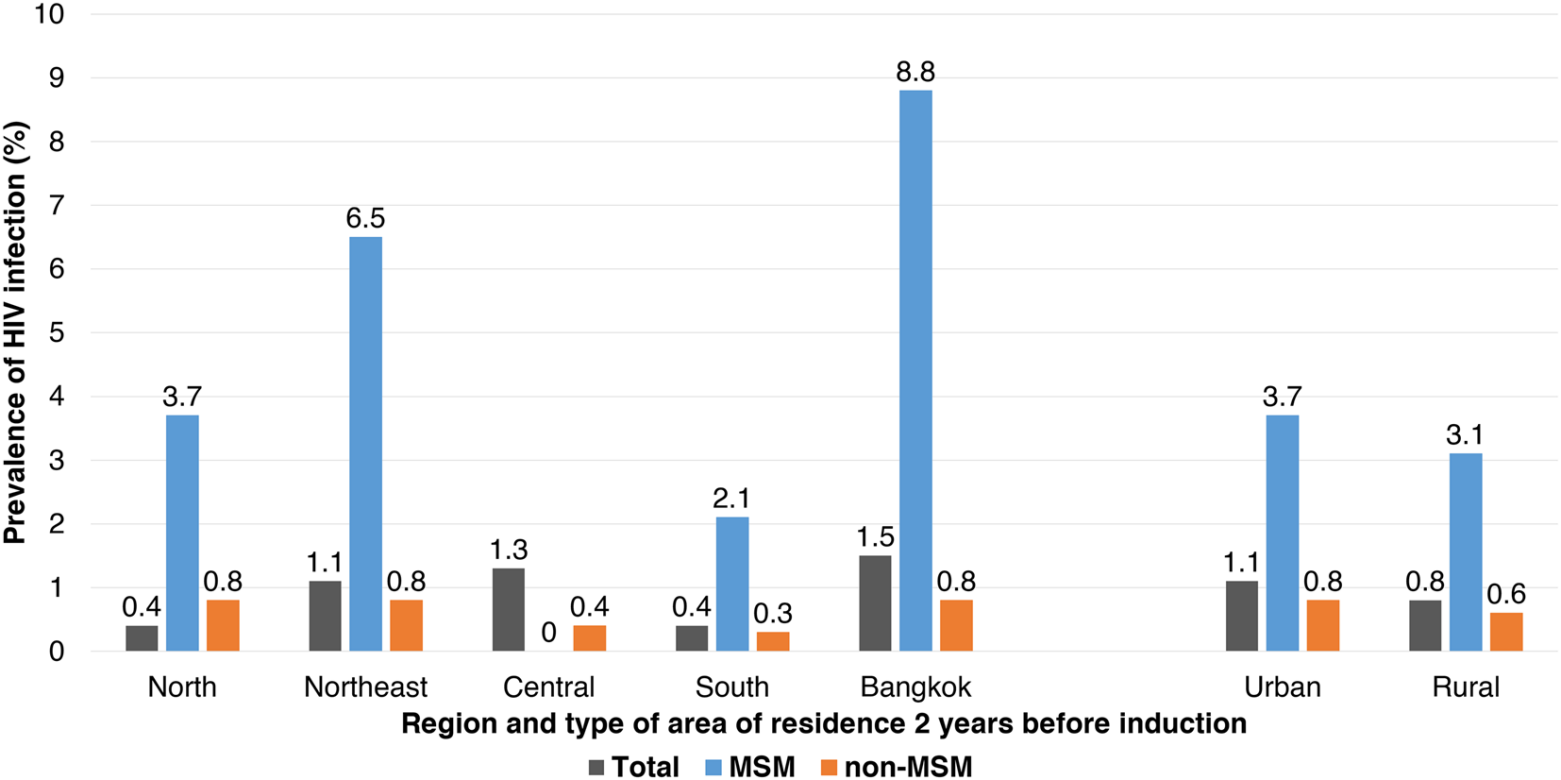
Prevalence of HIV infection among participants by region and type of area (rural vs. urban) of residence 2 years before induction.

A total of enrolled participants, 429 (10.1%, 95%CI; 9.1–10.9%) young Thai men reported a history of sex between men. Among the total HIV positive cases, 17 (38.6%) were identified to be MSM. HIV prevalence among MSM was 4.0% (95%CI; 2.1–5.8%), while it was 0.7% (95%CI; 0.4.1–0.9%) among non-MSM participants. Of 429 MSM study participants, the prevalence of HIV infection by region of residence two years before induction was 8.8, 6.5, 3.7, 2.1, and 0% in Bangkok, northeast, north, south and central regions, respectively. The prevalence of HIV infection among MSM study participants residing in urban and rural areas was comparable at 3.7 and 3.1%, respectively (Fig. 1).

In terms of sexual preference among MSM participants, the prevalence of HIV infection was 0.9, 6.3, and 23.4% among those who preferred to have sex with females only, both male and female, and male only, respectively. In terms of sexual experience among MSM participants, the prevalence of HIV infection was 1.7 and 16.7% among bisexuals and exclusive homosexuals, respectively (Table 3).

**Table 3:** Prevalence of HIV infection among men who have sex with men (MSM) and non-MSM participants in 2018.

Characteristics	MSM		Non-MSM	
	Total	HIV + (%)	Total	HIV + (%)
**Total**	429	17 (4.0)	3826	26 (0.7)
**Age (years)**	21.6 ± 1.3	21.6 ± 1.0	21.6 ± 1.2	21.5 ± 1.0
**Living pattern**				
Parents	286	11 (3.8)	2764	17 (0.6)
Lover/wife	73	1 (1.4)	602	6 (1.0)
Relatives and friends	37	1 (2.7)	281	2 (0.7)
Alone	13	2 (15.4)	85	1 (1.2)
**Region of residence 2 years before induction**				
North	108	4 (3.7)	1073	9 (0.8)
Northeast	123	8 (6.5)	1114	9 (0.8)
Central	43	0 (0.0)	488	2 (0.4)
South	97	2 (2.1)	778	2 (0.3)
Bangkok	34	3 (8.8)	262	2 (0.8)
**Place of residence**				
Urban	162	6 (3.7)	1576	13 (0.8)
Rural	128	4 (3.1)	1139	7 (0.6)
**Occupation**				
Employed	200	12 (6.0)	1866	17 (0.9)
Student	50	1 (2.0)	507	2 (0.4)
Agricultural	61	0 (0.0)	489	3 (0.6)
Unemployed	53	2 (3.8)	470	2 (0.4)
**Education attainment**				
No formal to grade 6	113	2 (1.8)	732	8 (1.1)
Grade 7–grade 12	208	9 (4.3)	1811	10 (0.6)
Vocational and diploma	57	4 (7.8)	866	5 (0.6)
Bachelor’s degree	48	2 (4.2)	385	3 (0.8)
**Marital status**				
Single	302	14 (4.6)	2754	19 (0.7)
Married	115	3 (2.6)	1018	7 (0.7)
**Sexual experience**				
Exclusive heterosexual	–	–	3660	24 (0.7)
Exclusive homosexual	66	11 (16.7)	–	–
Bisexual	361	6 (1.7)	–	–
**Sexual experience**				
Female only	339	3 (0.9)	3782	24 (0.6)
Male only	47	11 (23.4)	2	0 (0.0)
Both male and female	32	2 (6.3)	11	2 (18.2)

### Risk factors for HIV infection among young Thai men

Univariate and multivariate analyses were performed to identify the significant risk factors for HIV infection (Table 4). The independent risk factors for HIV infection among young men included having sex with men (Adjusted odds ratio (AOR): 4.7; 95% CI: 2.3–9.6), having a history of STI (AOR: 2.6; 95% CI: 1.1–6.3) and having a history of sex in exchange for gifts/money (AOR: 3.0; 1.2–7.5). Having a history of sex with a FSW (AOR: 0.5; 95% CI: 0.2–1.2) was found not to constitute a significant risk factor for HIV infection among the participants.

**Table 4:** Univariate and multivariate analysis of risk factors for HIV infection among the newly inducted army conscripts that entered into military service in November 2018.

Characteristics	Total	HIV + (%)	Crude Odds Ratio	Adjusted Odds Ratio
			**(95% CI)**	**(95% CI)**
**Age (years)**				
Mean (± SD)	21.6 ± 1.2	21.5 ± 1.0	1.1 (0.6–2.1)	0.8 (0.5–1.3)
**Living pattern**				
With parents	3366	29 (0.9)	1	
With lover/wife	673	7 (1.0)	1.2 (0.7–2.1)	
With relatives and friends	338	3 (0.9)	1.0 (0.5–2.2)	
Alone	103	3 (2.9)	3.4 (1.6–7.6)	
**Region of residence 2 years before induction**				
Central	564	2 (0.4)	1	
Northern	1284	14 (1.1)	3.1 (0.7–13.7)	
Northeast	1314	17 (1.3)	3.7 (0.9–16.0)	
Southern	979	4 (0.4)	1.2 (0.2–6.3)	
Bangkok	324	5 (1.5)	4.4 (0.9–22.8)	
**Place of residence**				
Rural	1361	11 (0.8)	1	
Urban	1896	20 (1.1)	1.3 (0.6–2.7)	
**Occupation**				
Student	628	3 (0.5)	1	
Agriculture	609	3 (0.5)	1.0 (0.2–5.1)	
Employee	2195	30 (1.4)	2.9 (0.9–9.5)	
Unemployed	587	4 (0.7)	1.4 (0.3–6.4)	
**Single**	3403	34 (1.0)	1.1 (0.6–2.3)	
**Education attainment**				
No formal to grade 6	906	10 (1.10)	1.3 (0.5–3.1)	1.0 (0.4–2.6)
Grade 7–grade 12	2165	20 (0.9)	1.0 (0.5–2.3)	0.8 (0.3–1.7)
Vocational and diploma	1014	9 (0.9)	1	1
Bachelor’s degree	492	5 (1.0)	1.2 (0.4–3.4)	1.6 (0.3–7.7)
**History of injecting drug use**				
No	4126	39 (1.0)	1	
Yes	220	4 (1.8)	1.9 (0.7–5.5)	
**History of non-injecting drug use**				
No	2460	21 (0.9)	1	
Yes	1996	20 (1.0)	1.2 (0.6–2.2)	
**History of sex with a female sex worker**				
No	3269	35 (1.1)	1	1
Yes	889	6 (0.7)	0.6 (0.3–1.5)	0.5 (0.2–1.2)
**Number of lifetime sex partner**				
Mean, ± SD	24.7 ± 39.3	29.1 ± 41.0	1.0 (1.0–1.0)	
**History of sex with another man**				
No	3826	26 (0.7)	1	1
Yes	429	17 (4.0)	6.0 (3.3–11.2)	4.7 (2.3–9.6)
**Sexual experience**				
Exclusively heterosexual	3660	24 (0.7)	1	
Exclusively homosexual	66	11 (16.7)	30.3 (14.2–69.9)	
Bisexual	361	6 (1.7)	2.5 (1.0–6.3)	
**Sexual preference/desire**				
Female only	4455	28 (0.6)	1	
Male only	58	11 (19.0)	37.0 (17.4–78.7)	
Bothe male and female	50	4 (8.0)	13.7 (4.6–40.8)	
**History of sexual transmitted infection**				
No	3846	34 (0.9)	1	1
Yes	231	7 (3.0)	3.5 (1.5–8.0)	2.6 (1.1–6.3)
**History of sex in exchange for gifts/money**				
No	4016	34 (0.9)	1	1
Yes	231	8 (3.5)	4.2 (1.9–9.2)	3.0 (1.2–7.5)
**History of sexual coercion**				
No	4085	38 (0.9)	1	
Yes	162	5 (3.1)	3.4 (1.3–8.8)	

The number of HIV + individuals and in the total column in each characteristic was based the response of study participants.

## Discussion

Our data exhibited patterns of sexual behavior and risk factors for HIV infection among young Thai men from army training units in selected five provinces in November 2018. Because the conscription process using a lottery system was conducted at the district level of the conscripts’ home province, our study population could to some extent represent young Thai men from both rural and urban areas^3,7^. We determined that the prevalence of HIV infection among the newly inducted army conscripts from the training units in selected provinces in 2018 was 1.0% (95% CI: 0.7–1.3%), which was comparable to the national HIV prevalence among those participating in the surveillance program for HIV among military conscripts indicating a HIV prevalence of 0.9% (336 of 38,225) (95%CI: 0.8–1.0%) during the same period. The prevalence of HIV infection among RTA new conscripts stabilized at 0.5% since 2004. This was significantly higher than the previous data though still below the 1% mark^2,3,8^.

We also found that though the prevalence of HIV infection among study participants residing in urban and rural area was comparable. In Thailand, effective HIV prevention intervention programs among MSM have been established especially in venue-based urban settings^9–12^. Our data suggested that monitoring and prevention measures should also be conducted in the rural areas that usually have more limited resources, as well as more limited ability to access health and social supports in the community^13^.

The present study illustrated that the proportion of reported cases of history of sex with another man was 10.1% (95%CI; 9.1–11.0%), while it was 7.9% (95%CI; 7.6–8.1%) from 2010 to 2011^7^. Our study reported that MSM played a major role in acquiring HIV infection among the participants. From the total 44 identified HIV infected cases, 17 (38.6%) reported having sex with another man. The prevalence of HIV infection among Thai MSM in our present study was 4.0% which was higher than that among young Thai MSM from 2010 to 2011 almost two fold (2.6%)^7^. In terms of geographical region resided in two years before conscription, HIV prevalence among MSM participants was significantly high in Bangkok (8.8%) and the northeast region (6.5%) which was higher than that among young Thai MSM from 2010 to 2011 (4.7% in Bangkok and 2.2% in the northeast)^7^. Nevertheless, the recent study at the Silom Community Clinic in Bangkok reported that HIV declined among Thai MSM between 2010 and 2018 because of PrEP implementation and has expanded substantially in Bangkok^9^. However, our finding reported that approximately 15% of young Thai MSM knew about PrEP, only 1.4% PrEP. Additionally, a low proportion of consistent condom use with a male partner was found, less than 40%. Our data suggested that effective HIV prevention interventions for MSM in Thailand including PrEP and condom use should be implemented both in rural and urban areas, especially in Bangkok and the northeast.

One of the significant findings related to MSM sexual activity from the present study was that the majority (81.1%) of those having sex with another man reported having exclusively heterosexual desire comparable with the finding in the related study from 2010 to 2011^7,14^. Related studies in China and the UK reported that the proportion of MSM with heterosexual desire were 1.6% and 22.0%, respectively^15–17^. In addition, we found an increasing proportion of bisexual activity among MSM of 60.6% from 2010 to 2011^7^ to 84.5% in our present study.

Among all participants, 5.4% had sex in exchange for gifts/money, while it was 14.5% among MSM study participants. We found that having sex in exchange for gifts/money was an independent risk factors for HIV infection. This behavior in these young men population may enhance the probability of HIV transmission from the older HIV postitive population.

The proportion of young Thai men reporting a history of sex with a FSW continued to decline since 2010 to 2011 (34.6%) to 21.4% in the present study, and was not associated with HIV infection. Additionally, the reported rate of consistent condom use during sex with a FSW was relatively high at 83.7%.

Because our study participants were homogenous in terms of age distribution; thus, we were unable to explore the effect of age on acquiring HIV infection in this study. The study employed a cross-sectional design, and as such, the results may have limited explanations regarding temporal relationships regarding both the exposure and outcome. Additionally, limitations were encountered related to the use of self-administrated questionnaires that might have provided limited answers especially regarding issues concerning sensitive matters.

## Conclusion

In conclusion, we reported the epidemiological data regarding HIV prevalence and related risk factors among randomly selected newly inducted army conscripts entering military service in five regions nationwide in Thailand. An increasing prevalence of HIV was found among young Thai men in 2018. The risk factors of HIV infection included having sex with another man, a history of sexual transmitted infection, and history of sex in exchange for gifts/money. HIV prevention programs including PrEP in Thailand should be emphasized among MSM in both rural and urban areas.

## Data Availability

The datasets generated during and/or analyzed during the current study are not publicly available because the data sets containssensitive identifying information including HIV status. Thus, due to ethical restrictions concerning the data sets, they are available from the corresponding author on reasonable request.

## Abbreviations

HIV: Human immunodeficiency virus
AIDS: Acquired immunodeficiency syndrome
MSM: Men who have sex with men
FSW: Female sex workers
STIs: Sexually transmitted infections
PrEP: Pre-exposure prophylaxis
PEP: Post-exposure prophylaxis
CI: Confidence Interval
